# Effects of Neuromuscular Electrical Stimulation with Gastrocnemius Strengthening on Foot Morphology in Stroke Patients: A Randomized Controlled Trial

**DOI:** 10.3390/healthcare12070777

**Published:** 2024-04-03

**Authors:** Yusik Choi, Sooyong Lee, Minhyuk Kim, Woonam Chang

**Affiliations:** 1 Department of Physical Therapy, Seoul Metropolitan Seonam Hospital, Yangcheon-gu, Seoul 08049, Republic of Korea; cus6208@naver.com; 2 Department of Physical Therapy, Severance Rehabilitation Hospital, Yonsei University, Seodaemun-gu, Seoul 03722, Republic of Korea; ywlee2000213017@hanmail.net; 3 Department of Physical Therapy, Graduate School, Yong In University, Yongin-si 17092, Gyeonggi-do, Republic of Korea; mmhh333@hanmail.net; 4 Department of Physical Therapy, College of Health & Welfare Science, Yong In University, Yongin-si 17092, Gyeonggi-do, Republic of Korea

**Keywords:** gastrocnemius, foot morphology, neuromuscular electrical stimulation, stroke

## Abstract

This study aimed to investigate the effects of neuromuscular electrical stimulation (NMES) with gastrocnemius (GCM) strength exercise on foot morphology in patients with stroke. Herein, 31 patients with chronic stroke meeting the study criteria were enrolled and divided into two groups; 16 patients were randomized to the gastrocnemius neuromuscular electrical stimulation (GCMNMES) group, and 15 patients to the conventional neuromuscular electrical stimulation (CNMES) group. The GCMNMES group conducted GCM-strengthening exercise with NMES. CNMES group conducted NMES at paretic tibialis anterior muscle with ankle dorsiflexion movement. These patients underwent therapeutic interventions lasting 30 min/session, five times a week for 4 weeks. To analyze changes in foot morphology, 3D foot scanning was used, while a foot-pressure measurement device was used to evaluate foot pressure and weight-bearing area. In an intra-group comparison of 3D-foot-scanning results, the experimental group showed significant changes in longitudinal arch angle (*p* < 0.05), medial longitudinal arch angle (MLAA) (*p* < 0.01), transverse arch angle (TAA) (*p* < 0.01), rearfoot angle (RA) (*p* < 0.05), foot length (FL) (*p* < 0.05), foot width (FW) (*p* < 0.05), and arch height index (AHI) (*p* < 0.01) of the paretic side and in TAA (*p* < 0.05) and AHI (*p* < 0.05) of the non-paretic side. The CNMES group showed significant changes in TAA (*p* < 0.05) and FW (*p* < 0.05) of the paretic side and TAA (*p* < 0.05) and AHI (*p* < 0.05) of the non-paretic side. An inter-group comparison showed significant differences in MLAA (*p* < 0.05) and RA (*p* < 0.05) of the paretic side. In an intra-group comparison of foot pressure assessment, the experimental group showed significant differences in footprint area (FPA) (*p* < 0.05) of the paretic side and FPA symmetry (*p* < 0.05). The CNMES group showed a significant difference in only FPA symmetry (*p* < 0.05). An inter-group comparison showed no significant difference between the two groups (*p* < 0.05). Thus, NMES with GCM-strengthening exercises yielded positive effects on foot morphology in patients with stroke.

## 1. Introduction

Stroke is a neurological condition characterized by a reduced blood supply to the brain tissue [1], resulting in paralysis of part or all of the body, limiting physical activity and social participation [2]. Gait impairment accounts for the largest proportion of physical activity limitations in stroke patients [3], and is characterized by muscle weakness, foot drop, spasticity, and foot deformity [4]. Especially, the foot has medial longitudinal and transverse arches, with the arch functioning as a kinetic chain from the calf to the front of the foot, controlling the degree of freedom of movement [5,6] and distributing the load by connecting the front of the foot to the back and the inside to the outside [7,8]. In stroke patients, decreased muscle strength and sensory deficits caused by the paretic side’s spasticity increase [9], and these changes result in a limited range of joint motion. In particular, the abnormal foot shape caused by spasticity becomes excessively supinated, which reduces the area of the foot in contact with the ground and adversely affects balance and gait [10].

Generally, the ankle joint is pivotal for balance and postural control during standing and walking [11] and contributes to sensory feedback crucial for maintaining proper posture through the soles of the feet [12]. The gastrocnemius muscle (GCM) plays a vital role in maintaining balance by keeping the feet in close contact with the ground during walking [13]. As a result, the weakening of this muscle can result in balance deficits and an increased risk of falls [14]. Moreover, inadequate lengths of the GCM, soleus, and Achilles tendons restrict foot movement and disrupt the forward movement of the tibia relative to the talus [15]. 

To address these issues, various approaches utilizing NMES have been shown to have a positive impact on gait and balance [16,17,18]. In particular, a study combining NMES with conventional physiotherapy was reported to be effective in reducing spasticity and strengthening ankle plantar flexors and improving exercise recovery [19]. A study conducted by Sabut et al. [20] reported that the combination of NMES and general physical therapy was effective in reducing stiffness and improving ankle dorsi-flexion strength and motor recovery in stroke patients. In addition, recent studies have reported that combining NMES with active stretching of the ankle plantar flexors significantly reduces spasticity and increases joint range of motion [21,22]. These results suggest that NMES treatment may be more effective when combined with active movement than NMES treatment alone [23,24]. 

Previous studies have shown that tibialis-anterior NMES application improves ankle weakness [25,26]. However, the physical symptoms seen in stroke patients include foot drop and muscle weakness due to stiffness, which is not caused by weakness of the tibialis anterior muscle but rather by weakness of the GCM muscle, resulting in various consequences such as restricted ankle-joint movement, foot arch deformity, genu recurvatum (back knee), balance problems, and slow gait speed [27]. However, only a few studies have applied NMES to GCM. Therefore, in this study, a new intervention, NMES with GCM-strengthening training, was designed, and its effect on foot morphology in patients with stroke was investigated. The significance of this research is that it suggests a new treatment that could change the foot morphology, which can cause problems for stroke patients.

## 2. Materials and Methods

### 2.1. Study Design

**Figure 1:**
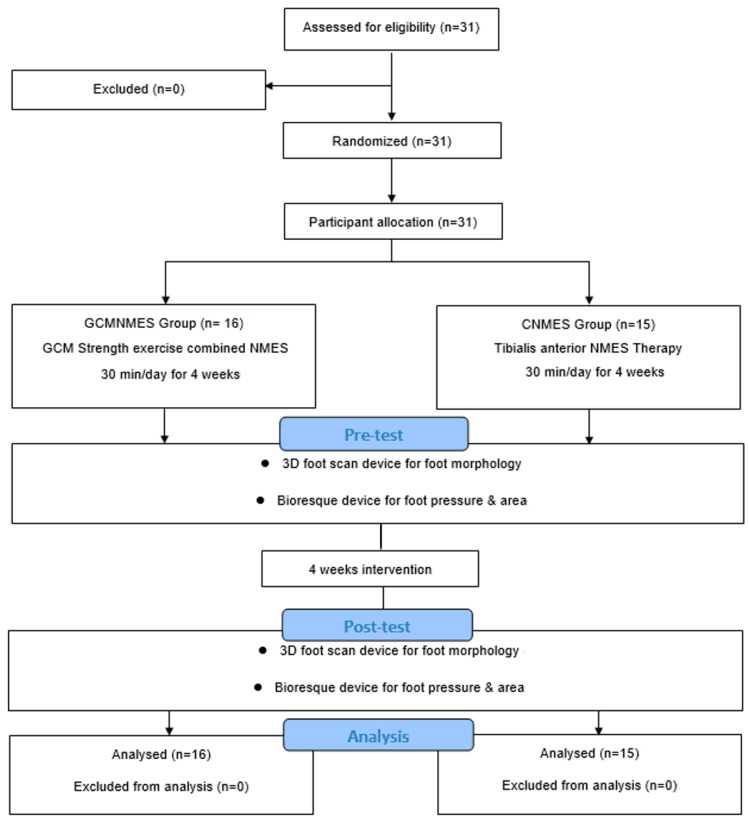
Visual representation following the CONSORT guidelines for the participant-enrollment process in this study. Abbreviations: GCM, gastrocnemius muscle; NMES, neuromuscular electrical stimulation.

This study employed a randomized controlled trial design with a parallel-group allocation ratio of 1:1. Participants were assigned to either the NMES with GCM-strengthening exercise (GCMNMES) or the conventional NMES therapy (CNMES) group through randomization. The software program “Microsoft Excel (version 16.0)” (Microsoft Corp., Redmond, WA, USA) was employed for this purpose. The study duration was 4 weeks, including a pre-evaluation, and a post-evaluation was conducted 1 month later. Out of a total of 100 patients with stroke from a single medical institution, 31 patients who met the inclusion criteria were randomly assigned to one of two groups, the GCMNMES group or the CNMES group, using a random number generator (selected as 1 and 2), specifically the “RANDBETWEEN” function in Microsoft Excel. This freely available software allows for both simple and blocked random allocation. To enhance the objectivity of the assessments, this study was double blinded to both researchers and subjects. The study adhered to the Consolidated Standards of Reporting Trials (CONSORT) guidelines [28]. The framework of this study is shown in Figure 1.

### 2.2. Ethical Considerations

This study adhered to the principles outlined in the Declaration of Helsinki [29]. Informed consent was obtained from all participants, and ethical approval was obtained from the institutional review board of Yong In University (approval ID: 2212-HSR-283-2). International clinical trial registration numbers are issued by the Clinical Research information Service (CRIS), which is registered with the World Health Organization (WHO) International Clinical Trials Registry Platform (ICTRP) (approval ID: KCT0009188).

### 2.3. Participants

The sample size was determined using G*Power software (version 3.1.9.6; Heinrich Heine University, Düsseldorf, Germany) under the following configurations: *t*-tests, a calculated size effect (1.43) based on a previous study [30,31,32], a significance level (α) of 0.05, and a desired statistical power of 0.80. The determined sample size was 31 participants, evenly distributed, with 16 individuals in the study group and 15 individuals in the CNMES group.

Patients were recruited from the Dae Jeon Rehabilitation Hospital, Daejeon City, Republic of Korea. The inclusion criteria were as follows: Chronic stroke patients; Korean-Mini-Mental Status Examination (K-MMSE) score >24 points; Ability to walking independently for 10 meters or more with or without aids; absence of joint contractures, pain, or musculoskeletal fractures; and an ability to comprehend the purpose of this study and participate voluntarily [33]. The exclusion criteria were: Inability to stand due to restricted ankle joint range of motion, presence of metal objects resulting from calf or ankle surgery, presence of a cardiac pacemaker or history of heart disease, and other medical histories (venous and arterial thrombosis, thrombophlebitis) [21]. A total of 31 stroke patients who met the inclusion criteria were enrolled in the study.

Participants were randomly assigned to either the GCMNMES group or the CNMES group. Informed consent was obtained from all participants in accordance with the institutional review board guidelines. The study was double blinded to both the researchers and subjects. The study involved 31 stroke patients recruited from January to March 2023 with pre- and post-assessments after a 4-week intervention period. 

The study involved outpatients attending a healthcare center, and both the GCMNMES and CNMES groups received routine rehabilitation treatment (range of motion exercises, strength training, balance, and gait training) two or three times a week. All patients had a chronic stroke with an onset date of >60 months, and no medication directly affected the intervention.

### 2.4. Intervention

#### 2.4.1. GCMNMES

GCMNMES was conducted using Microstim (Medel GmbH, Hamburg, Germany) and an incline board (StrongTek Professional Wooden Incline Board; Slantek, Sugar Land, TX, USA). Electrodes were attached to the muscle fibers located 2 cm laterally from the center of the paretic GCM, with the electrode aligned parallel to the muscle fibers. Following the treatment, intermittent low-frequency alternating stimulation was employed to minimize muscle fatigue [34], with monophasic rectangular waves applied at a pulse frequency of 35 pps for a pulse duration of 150–350 ms and stimulation and rest times (on–off ratio) of 5 s each [30]. The current intensity was adjusted to a range that induced muscle contraction in each participant, and the application time was set to 30 min.

**Figure 2:**
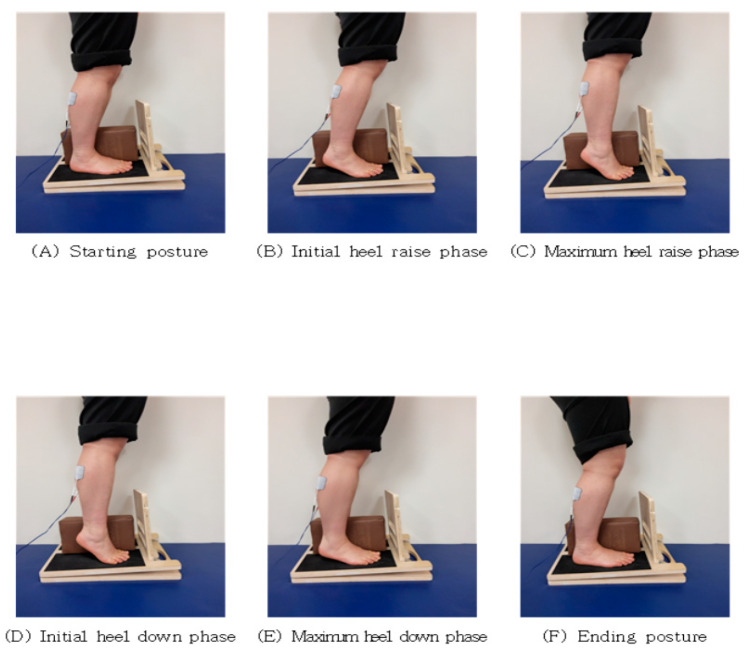
NMES with GCM-strengthening training.

GCMNMES exercises were adapted from the works of Hur [21], Choi and Chang [32], and Cheng et al. [35]. The starting position involved adopting a comfortable standing position, with the feet positioned on a basal plane at the width of the pelvis [36]. A reference point was provided to ensure the pelvis was in a neutral position [37]. Subsequently, patients were engaged in antigravity activities while standing [38]. To prevent excessive supination of the ankle joint and maintain consistent foot positioning, a square block measuring 280 mm × 150 mm × 90 mm was placed between the feet. The GCM-strengthening exercise involved raising and lowering the heel on an inclined board, with an adjustable angle (0°, 5°, 10°), to accommodate varying levels of difficulty based on the patient’s condition. This exercise was combined with NMES treatment and performed for 30 min (Figure 2). The specific sequences are detailed in Table 1. The intervention was conducted under researcher supervision in a safe environment with sidebars or tables set up for patients to hold onto to prevent the risk of falls.

#### 2.4.2. CNMES

In the CNMES group, NMES treatment was administered to the paretic tibialis anterior while participants were in a seated position for 30 min to deliver electrical stimulation. The electrodes were strategically placed at the posterior part of the paretic fibular head and the motor point of the tibialis anterior. Electrical stimulation was applied in monophasic rectangular waves at a pulse frequency of 35 pps, pulse duration of 150–350 ms, and both stimulation and rest times (on–off ratio) lasting 5 s each [30]. The permissible current level was adjusted to the intensity at which muscle contraction occurred at the electrodes attached to each participant. In the sitting position, ankle dorsiflexion movements were performed simultaneously with the soles of the feet in contact with the ground.

### 2.5. Outcome Measures

#### 2.5.1. Foot Morphology

**Figure 3:**
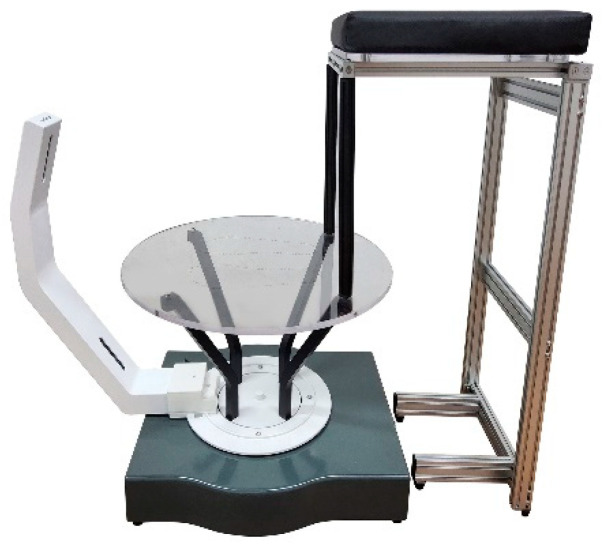
Foot 3D scanning.

Foot morphology was examined using foot 3D-scanning equipment (MeiACE Scan MS320A, Realdimension Inc., Daegu, Republic of Korea) (Figure 3). This equipment is capable of scanning foot shapes in a standing position with both feet supporting the body weight [39], and the scanner camera rotates 360° to generate 3D images of the participant’s feet. To obtain accurate 3D-surface data on bone locations, 14 markers were placed on both feet, and seven foot-shape data points were measured based on the 3D data. Various foot-shape data were analyzed, including longitudinal arch angle (LAA), medial longitudinal arch angle (MLAA), transverse arch angle (TAA), rearfoot angle (RA), foot length (FL), foot width (FW), and arch height index (AHI). Foot shape and pressure measurements were conducted both before and after the intervention. All physical and scan measurements of correlation were greater than or equal to 0.80, indicating high correlation. Most intraclass correlation coefficient values were above 0.95 [40,41]. 

#### 2.5.2. Foot Pressure

Foot pressure was evaluated using the BioRescue platform (BioRescue; RM Ingenierie, Marseille, France) comprising 1600 pressure sensors at a density of 1 per cm^2^, measuring 610 mm × 580 mm × 10 mm. This platform is designed to capture fine movements and body alignment in an upright position. During the evaluation, patients stood comfortably on a balance-measurement platform equipped with a safety bar (heel spacing of approximately 3 cm, two feet spread to approximately 30°). The patients were instructed to keep their eyes open, look straight ahead, and place both arms next to the trunk. The patients were required to maintain this posture for 10 s with their gaze fixed on the front, and the frequency (%) and area (mm^2^) of the foot pressure applied to the four measurement areas were measured. The equipment has an intra-rater reliability of 0.79 and inter-rater reliability of 0.92 [42].

### 2.6. Statistical Analysis

All data were analyzed using SPSS ver. 18.0 (IBM Corp., Armonk, NY, USA), a statistical program. Chi-square and independent *t*-tests were conducted to assess the homogeneity of the GCMNMES and CNMES groups. Independent *t*-tests were employed after ensuring homogeneity to identify differences in the dependent variables between the groups. The general characteristics of the participants were denoted as means and standard deviations using descriptive statistics, and the Shapiro–Wilk test was used to assess normality.

Statistical evaluation across all groups was performed by analyzing changes in foot shape and foot pressure. Paired *t*-tests were conducted to determine the significance levels before and after treatment within groups, while independent *t*-tests were performed for comparisons between groups. The measurement results of each item were presented as the mean ± standard deviation, with *p*-values < 0.05 considered statistically significant (α = 0.05). 

## 3. Results

### 3.1. General Characteristics of Study Subjects 

The demographic details of the participants are outlined in Table 2. No significant differences were observed between groups in sex, stroke type, paretic side, age, height, duration, MMSE-K score, and K-MBI score. Importantly, all participants successfully completed the training program and there were no dropouts.

### 3.2. Comparison of Foot Shape before and after Intervention

**Figure 4:**
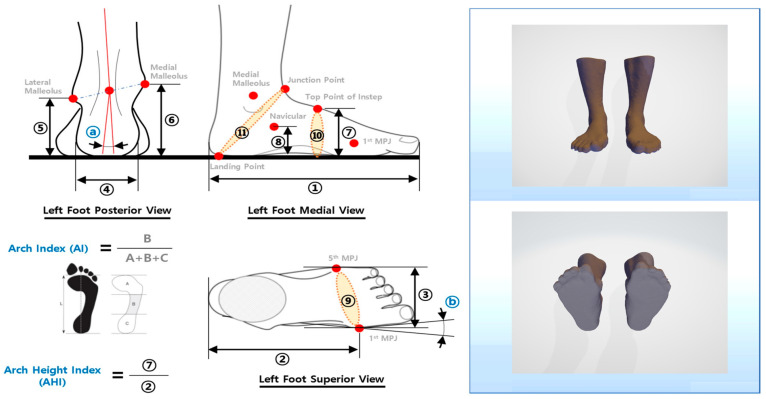
Foot measurement and analysis report.

The GCMNMES group showed a statistically significant decrease in paretic MLAA (*p* < 0.01), paretic TAA (*p* < 0.01), nonparetic TAA (*p* < 0.05), paretic RA (*p* < 0.05), paretic FL (*p* < 0.05), paretic FW (*p* < 0.05) and an increase in paretic LAA (*p* < 0.05), paretic AHI (*p* < 0.01) between pre-and post-test values. Conversely, the CNMES group showed a statistically significant decrease in paretic TAA (*p* < 0.05), nonparetic TAA (*p* < 0.05), paretic FW (*p* < 0.05) and an increase in nonparetic AHI (*p* < 0.05) between pre-and post-test values. Intergroup comparison between the GCMNMES and CNMES groups showed statistically significant differences in MLAA (*p* < 0.05) and RA (*p* < 0.05) (Table 3). Figure 4 visually provides the result of calculating the data value of each item using 3D-scanning equipment. 

### 3.3. Comparison of Foot Pressure before and after Intervention

**Figure 5:**
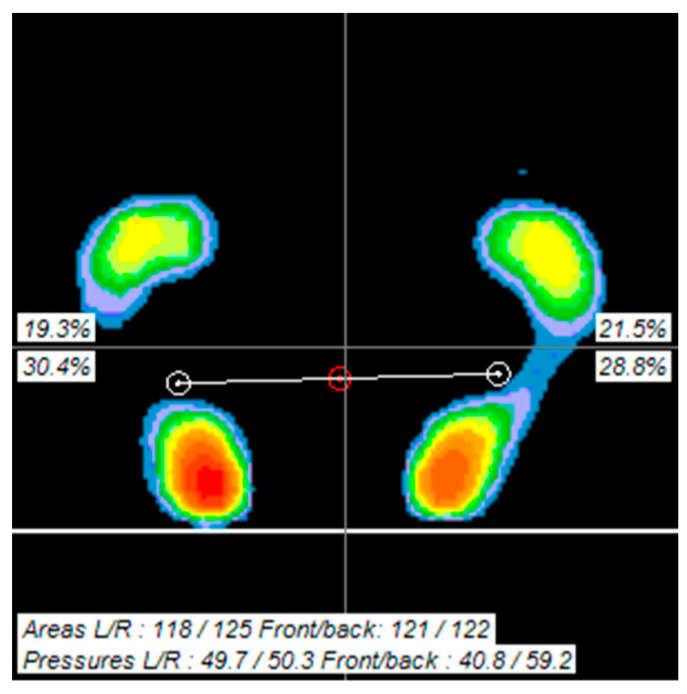
Footprint area and footprint pressure.

The GCMNMES group demonstrated a statistical decrease in nonparetic FPP (*p* < 0.05) and an increase in paretic footprint area (FPA) (*p* < 0.05), FPA symmetry (*p* < 0.05), paretic footprint pressure (FPP) (*p* < 0.01) between pre-and post-test values. In contrast, the CNMES group showed a statistically significant decrease in nonparetic FPP (*p* < 0.05) and an increase in FPA symmetry (*p* < 0.05) between pre-and post-test values (Table 4). Figure 5 is a visual representation of the footprint area and pressure changes measured using the BioRescue platform. 

## 4. Discussion

NMES has long been recognized as an effective approach for addressing balance and gait disorders in patients with stroke [20]. Repetitive electrical stimulation, for instance, has shown efficacy in increasing skeletal-muscle activity and strength, with observable improvements occurring after approximately 4 weeks of NMES treatment [43]. Bogataj et al. [44] reported that NMES applied to the thigh and ankle dorsiflexors of the paretic leg could enhance walking efficiency by promoting the effective movement of the knee and ankle joints. Moreover, previous research has consistently emphasized the positive impact of GCM strengthening via NMES on the balance and gait of patients with stroke, which is consistent with the findings of this study. 

### 4.1. Foot Morphology

In the intragroup comparison of paretic LAA, the GCMNMES group exhibited a statistically significant increase in LAA from 142.77° to 151.36° (*p* < 0.05). McPoil and Cornwall [45] categorized foot arches with LAA close to 90° as low arches and those with LAA close to 180° as high arches. These results support the findings of this study, indicating that the treatment intervention employed in this study can restore the foot arch by increasing the LAA and alleviating compensatory actions, such as excessive foot-pronation patterns, in patients with stroke.

In the intragroup comparison of paretic MLAA, the GCMNMES group exhibited a statistically significant decrease in MLAA from 137.23° to 131.38° (*p* < 0.01). Jang [46] conducted a navicular drop test to identify structural changes influencing foot shapes and reported statistically significant differences in drop values between the paretic and nonparetic sides in patients with stroke, with the paretic side exhibiting a drop of 6.3 mm and the nonparetic side showing 7.8 mm (*p* < 0.05). Additionally, the study found that the paretic side had a lower drop than the nonparetic side for various reasons [47]. Billis et al. [48] highlighted the importance of changes in navicular bone height in determining foot shapes, such as pronation and supination. They stated that a greater drop in the navicular bone corresponds to a higher tendency toward foot pronation. These findings were consistent with the LAA and MLAA measurements reported in the present study, where patients exhibited a low foot arch in the standing position. Chen et al. [49] measured the foot shape index of patients with stroke. They reported that the paretic foot shape index was higher than the non-paretic foot shape index, indicating a tendency toward foot pronation. This results in an increased contact surface of the foot via inward rotation of the knee to compensate for foot supination due to stiffness on the paretic side in the standing position. The angle of the MLAA reduced by the intervention in this study implies a restoration of the foot arch, which in turn implies a reduction in foot pronation.

Welte et al. [50] established a strong correlation (*p* < 0.01) between spasticity and paretic heel-bone tilt, indicating that greater spasticity led to a lateral tilt of the paretic heel bone compared to the nonparetic heel bone. Regarding the intragroup comparison of the paretic RA, the GCMNMES group showed a decrease from 7.45° to 4.25° (*p* < 0.05).

Jung et al. [51] reported a significant correlation between MLAA, RA, and the height of the navicular bone (*p* < 0.001). In a study by Jang [46] involving patients with stroke, the inclination of the nonparetic heel bone, measured in the standing position, was 0.22° ± 4.5°, close to a right angle. The inclination of the paretic heel bone was −1.51° ± 1.94°, indicating a slight inward tilt. Therefore, changing MLAA and RA in the standing position can influence foot shape. The results of this study suggest that a decrease in the paretic MLAA (*p* < 0.01) and RA (*p* < 0.05) can contribute to an increase in paretic arch height and the restoration of vertical alignment of the heel bone.

### 4.2. Foot Pressure 

According to the foot-pressure changes observed in this study, the weight-bearing area of the foot increased significantly from 95.51 mm^2^ before the intervention to 107.42 mm^2^ after the intervention on the paretic side in the GCMNMES group (*p* < 0.05). The symmetry of the foot area also increased significantly from 0.16 to 0.22 in the GCMNMES group (*p* < 0.05), and it increased from 0.17 to 0.19 in the CNMES group (*p* < 0.05). This study supports the hypothesis that the intervention described herein, which leads to the recovery of the FPA and symmetry, is effective in stabilizing the basal plane and restoring balance in patients with stroke.

Previous studies have suggested that changes in the length of the GCM muscle may affect balance by increasing the ankle-joint range of motion and shifting the pressure point of the foot posteriorly [31,39]. Therefore, we believe that the increase in weight bearing on the paretic side led to changes in the MLAA (*p* < 0.01) and RA (*p* < 0.05) in this study that increased paretic AHI (*p* < 0.01), decreased the paretic FL (*p* < 0.05), and increased paretic FW (*p* < 0.05).

### 4.3. Strengths and Limitations 

NMES with GCM-strengthening training demonstrated a positive effect on foot morphology in patients with stroke. The improvement in postural stability within the GCMNMES group was identified through the recovery of asymmetry in the FPA. An increase in the LAA of the foot shape and a decrease in the MLAA contributed to an increase in the paretic FPA ratio. Simultaneously, an increase in RA promoted inward torsion of the tibia relative to the talus, affecting the vertical alignment of the tibia.

GCM muscle strengthening plays an important role in balance and gait through reciprocal inhibition with TA muscle. Previous studies have shown that NMES can reduce spasticity [30,32]. Cheng et al. reported that NMES of the dorsiflexor activates eccentric muscle contraction of the plantar flexors [35], and several studies have shown that NMES of the GCM muscle can activate the postural extensor muscle through increased ankle-joint motion and heel shift of plantar pressure due to decreased plantar-flexor spasticity [21,31,39]. Therefore, the change in foot morphology through the intervention in this study suggests that NMES application of the GCM muscle and appropriate exercise methods may impact balance and gait.

Nevertheless, this study has some limitations. First, we could not control external factors other than the treatment intervention time, such as exercise programs. Second, although the number of study participants satisfied normality, the generalizability of our results may be limited. Third, the degree of leg stiffness and foot posture index were not considered in the selection criteria. Fourth, the intervention period was relatively short, lasting only 4 weeks. It is worth noting that while there are conflicting reports in the literature, muscle strength is generally reported to improve after 4 weeks of NMES treatment. However, some studies suggest that this effect may be observed when NMES treatment is applied for extended periods, such as 6–8 weeks [47]. Lastly, we did not measure the ground reaction force resulting from changes in foot morphology, preventing confirmation of the impact of these changes on the vertical alignment of the body.

## 5. Conclusions

GCMNMES effectively increased the basal plane, the area where the foot contacts the ground, via changes in foot morphology. Therefore, the strategy of NMES with GCM muscle-strengthening exercises presented in this study proved to be effective for improving foot morphology in patients with stroke.

## Figures and Tables

**Table 1:** Stage of exercise.

Stage	GCMNMES
A	Start with your knees bent at 5°.
B	Allow your heels to slowly lift off the floor for 5 s while the current is running.
C	Raise your heels as far as you can in a plantar flexion.
D	For 5 s without current, let your heels slowly touch the floor.
E	Slowly move your heels until they are fully on the floor.
F	Slowly return to a 5° knee bend.

**Table 2:** Clinical characteristics of participants (n = 31).

Variables	GCMNMES (n = 16)	CNMES (n = 15)	*x/t* ^2^	*p*
Sex (Male/Female)	10/6 (63/37)	8/7 (53/47)	0.271 ^b^	0.721
Stroke type (ICH/CI, %)	10/6 (63/37)	5/10 (33/67)	2.644	0.165
Paretic side (R/L, %)	9/7 (56/44)	8/7 (53/47)	0.032	1.000
Age (year)	46.19 ± 13.36 ^a^	63.93 ± 11.95	0.976 ^c^	0.336
Height (cm)	168.56 ± 7.84	165.27 ± 6.79	1.241	0.221
Duration (months)	68.10 ± 12.84	61.97 ± 8.36	1.562	0.133
MMSE-K (score)	44.25 ± 23.14	38.46 ± 34.07	0.568	0.585
K-MBI (score)	29.31 ± 1.31	26.93 ± 2.40	3.452	0.097

ICH: Intracranial Hemorrhage; CI: Cerebral Infarction; MMSE-K: Mini-mental State Examination-Korean; K-MBI: Korean-modified Barthel Index. ^a^ mean ± standard deviation, ^b^ Chi-square test, ^c^ independent *t*-test.

**Table 3:** Comparison of foot morphology.

Variables		GCMNMES Group (n = 16)			CNMES Group (n = 15)			*p*
		Pre-Test	Post-Test	Mean Difference (95% CI)	Pre-Test	Post-Test	Mean Difference (95% CI)	
LAA [◦]	*Paretic*	142.77 ± 6.69	151.36 ± 7.99 *^,a^	8.58 (6.9, 10.3)	147.23 ± 10.14	148.89 ± 8.40	1.67 (−1.9, 5.2)	0.148 ^b^
	*nonparetic*	144.79 ± 6.14	148.3 ± 11.79	3.55 (−1.0, 8.1)	148.91 ± 10.46	149.91 ± 10.46	0.15 (−3.5, 3.8)	0.390
MLAA [◦]	*Paretic*	137.23 ± 4.79	131.38 ± 5.84 **	−5.85 (−7.4, −4.3)	133.16 ± 7.02	132.03 ± 6.41	−1.13 (−3.2, 0.9)	0.049 *
	*nonparetic*	135.51 ± 5.41	134.0 ± 7.56	−1.47 (−4.1, 1.2)	132.11 ± 7.80	131.64 ± 5.77	−0.47 (−2.1, 1.2)	0.214
TAA [◦]	*Paretic*	116.08 ± 8.57	107.14 ± 9.23 **	−8.93 (−12.4, −5.4)	111.23 ± 6.02	105.03 ± 8.19 *	−6.02 (−9.0, −3.0)	0.394
	*nonparetic*	118.88 ± 7.16	112.19 ± 8.79 *	−6.69 (−10.3, −3.1)	114.63 ± 8.34	107.10 ± 7.62 *	−7.50 (−10.1, −4.9)	0.782
RA [◦]	*Paretic*	7.45 ± 8.78	4.25 ± 7.22 *	−3.03 (−5.3, −0.8)	5.00 ± 8.04	5.20 ± 10.31	0.20 (−1.9, 2.3)	0.013 *
	*nonparetic*	9.54 ± 7.04	7.45 ± 8.13	−2.09 (−4.2, −0.1)	8.00 ± 7.10	6.80 ± 8.90	−1.20 (−3.4, 1.0)	0.184
FL [mm]	*Paretic*	249.54 ± 13.02	247.02 ± 12.72 *	−2.52 (−3.9, −1.2)	243.38 ± 11.93	244.67 ± 10.80	1.29 (−0.7, 3.2)	0.381
	*nonparetic*	247.73 ± 12.72	247.48 ± 14.08	−0.24 (−1.6, 1.2)	246.45 ± 9.48	245.83 ± 9.38	−0.61 (−1.6, 0.4)	0.135
FW [mm]	*Paretic*	100.51 ± 7.08	98.89 ± 6.65 *	−1.61 (−2.7, −0.6)	97.03 ± 5.32	95.60 ± 4.98 *	−1.43 (−2.3, −0.6)	0.849
	*nonparetic*	101.26 ± 6.29	99.78 ± 6.12	−2.52 (−3.9, −1.2)	96.07 ± 5.12	94.49 ± 4.12	−1.59 (−2.7, −0.5)	0.925
AHI [index]	*Paretic*	0.39 ± 0.03	0.41 ± 0.04 **	0.02 (0.0, 0.0)	0.40 ± 0.04	0.41 ± 0.04	0.01 (0.0, 0.0)	0.318
	*nonparetic*	950.41 ± 0.04	0.43 ± 0.03 *	0.02 (0.0, 0.0)	0.41 ± 0.03	0.44 ± 0.04 *	0.03 (0.0, 0.0)	0.890

LAA: Longitudinal arch angle; MLAA: Medial longitudinal arch angle; TAA: Transverse arch angle; RA: Rearfoot angle; FL: Foot length; FW: Foot width; AHI: Arch height index. ^a^ Paired *t*-test, ^b^ Independent *t*-test. * *p* < 0.05, ** *p* < 0.01.

**Table 4:** Comparison of footprint area and pressure.

Variables		GCMNMES Group (n = 16)			CNMES Group (n = 15)			*p*
		Pre-Test	Post-Test	Mean Difference (95% CI)	Pre-Test	Post-Test	Mean Difference (95% CI)	
FPA [mm^2^]	*paretic*	95.51 ± 23.04	107.42 ± 22.23 *^,a^	11.91 (6.4, 17.4)	107.44 ± 24.00	106.65 ± 24.65	−1.24 (−5.9, 3.5)	0.236 ^b^
	*nonparetic*	111.69 ± 12.06	112.93 ± 17.66	0.79 (−4.9, 6.5)	113.15 ± 25.56	118.27 ± 21.03	5.13 (−0.4, 10.6)	0.706
	*symmetry*	0.16 ± 0.16	0.22 ± 0.30 *	0.05 (−0.0, 0.1)	0.17 ± 0.17	0.19 ± 0.15 *	0.02 (−0.0, 0.1)	0.481
FPP [%]	*paretic*	41.80 ± 7.72	46.37 ± 10.80 **	2.59 (−1.7, 6.9)	49.46 ± 8.29	52.06 ± 9.35	4.58 (1.07, 8.1)	0.627
	*nonparetic*	58.22 ± 6.72	53.83 ± 10.80 *	−3.22 (−6.9, 0.5)	50.54 ± 8.15	47.32 ± 8.23 *	−4.60 (−8.1, −1.1)	0.712
	*symmetry*	0.26 ± 0.22	0.33 ± 0.37	0.07 (−0.1, 0.2)	0.29 ± 0.18	0.31 ± 0.19	0.02 (−0.0, 0.1)	0.611

FPA: Footprint area; FPP: Footprint pressure. ^a^ Paired *t*-test, ^b^ Independent *t*-test, * *p* < 0.05, ** *p* < 0.01.

## Data Availability

Data are available upon requesting to the corresponding author, Woonam Chang.
